# Is Scale-Up of Community Mobilisation among Sex Workers Really Possible in Complex Urban Environments? The Case of Mumbai, India

**DOI:** 10.1371/journal.pone.0121014

**Published:** 2015-03-26

**Authors:** Anine Kongelf, Sunita V. S. Bandewar, Shalini Bharat, Martine Collumbien

**Affiliations:** 1 London School of Hygiene and Tropical Medicine, 15–17 Tavistock Place, London United Kingdom; 2 Independent Senior Researcher in Global Health, Bioethics and Program Evaluation, C-5, Mantri Avenue—I, Panchavati, Pashan Rd, Pune, India; 3 School of Health Systems Studies, Tata Institute of Social Sciences, Mumbai, India; University of Illinois at Chicago, UNITED STATES

## Abstract

**Background:**

In the last decade, community mobilisation (CM) interventions targeting female sex workers (FSWs) have been scaled-up in India’s national response to the HIV epidemic. This included the Bill and Melinda Gates Foundation’s Avahan programme which adopted a business approach to plan and manage implementation at scale. With the focus of evaluation efforts on measuring effectiveness and health impacts there has been little analysis thus far of the interaction of the CM interventions with the sex work industry in complex urban environments.

**Methods and Findings:**

Between March and July 2012 semi-structured, in-depth interviews and focus group discussions were conducted with 63 HIV intervention implementers, to explore challenges of HIV prevention among FSWs in Mumbai. A thematic analysis identified contextual factors that impact CM implementation. Large-scale interventions are not only impacted by, but were shown to shape the dynamic social context. Registration practices and programme monitoring were experienced as stigmatising, reflected in shifting client preferences towards women not disclosing as ‘sex workers’. This combined with urban redevelopment and gentrification of traditional red light areas, forcing dispersal and more ‘hidden’ ways of solicitation, further challenging outreach and collectivisation. Participants reported that brothel owners and ‘pimps’ continued to restrict access to sex workers and the heterogeneous ‘community’ of FSWs remains fragmented with high levels of mobility. Stakeholder engagement was poor and mobilising around HIV prevention not compelling. Interventions largely failed to respond to community needs as strong target-orientation skewed activities towards those most easily measured and reported.

**Conclusion:**

Large-scale interventions have been impacted by and contributed to an increasingly complex sex work environment in Mumbai, challenging outreach and mobilisation efforts. Sex workers remain a vulnerable and disempowered group needing continued support and more comprehensive services.

## Introduction

The recognition that factors in the broader social context constrain an individual’s ability to control the risk of HIV transmission has led to more complex HIV prevention models [1,2,3,4]. As part of a more comprehensive response, structural interventions attempting to alter the social environment that produces HIV-risk complement individual behavioural change interventions [3,5]. Community mobilisation (CM) has been seen as one such structural strategy for interventions targeting sex workers, with a focus on creating enabling environments and strengthening social norms that support individual sex workers’ agency [3,5,6]. The National AIDS Control Programme (NACP) of India pronounced community mobilisation as a central tenet of comprehensive sex worker programmes [7]. The appeal of CM is often traced back to the success of the ‘Sonagachi Project’ in Kolkata, West Bengal, referred to as the model example of a community-led intervention among female sex workers (FSWs) [8,9,10,11]. By 1999 HIV prevalence among FSWs reached 50–90% in Mumbai and other major cities in India, in sharp contrast to Kolkata, where rapidly increased levels of condom use (to 90%) kept HIV prevalence to around 11% [12].

The expectations raised by Sonagachi regarding the potential of mobilizing marginalised populations to reduce HIV transmission struck a chord with the leadership of Avahan, the India Aids Initiative funded by the Bill and Melinda Gates Foundation [13]. Launched in 2003 this flagship programme aimed to build an HIV prevention model ‘at scale’ in the six states most affected by HIV. The quick scale-up of Avahan’s standardised service package was implemented through 134 non-governmental organisations (NGOs) across 83 districts including the delivery of peer-based education with persistent focus on condom promotion and distribution and enhanced access and uptake of STI screening and treatment [14]. Use of monitoring data was institutionalised at all levels of the organization, including innovative tools to help outreach workers reach their targets [15]. Early implementation challenges reinforced the importance of engaging sex workers in the interventions [16]. Hence promoting community mobilization and investing in core capacity building of community based organizations (CBOs) with the aim of shifting ownership of the programme to the community became Avahan’s trademark ambition [17].

Massive investment in monitoring and evaluation, and a focus on effectiveness in reaching ‘saturation levels’ of prevention coverage made measuring tangible results a central need [18]. This required operationalising community mobilisation, a ‘social change’ intervention into health science frameworks in order to demonstrate epidemiologically observable effects [5]. Avahan developed a goal-based linear cause-effect evaluation framework [10] to link programme inputs to the desired programme outcomes: increased condom use and STI treatment seeking as well as adoption of positive behavioural norms. The theory of change hypothesises that ‘meaningful participation’ in high risk group intervention (HRGI), structural intervention (SI) and organisational development (OD) activities will lead to community mobilisation processes (identification with other sex workers, collectivisation and taking ownership of the intervention). This in turn will lead to improved programme outcomes, and eventually a sustained HIV prevention impact [10].

The HRGI activities are delivered by peer educators (PEs) and outreach workers (ORWs) and include one-to-one behaviour change communications; referral to clinical services, condom provision; and community group meetings in ‘safe spaces’[10]. They constitute the core of the ‘standard package’ of interventions as set out in the Avahan Common Minimum Program (CMP)[19]. With a focus on individual behaviour change, the indicators are monitored against targets via the Management Information Systems (MIS), the guide for monitoring Avahan’s implementation[19]. Programme monitoring indicators of ‘CM and Enabling Environment’ that measure progress on ‘Structural Intervention’ (SI) activities addressing FSWs legal and economic vulnerability were more challenging to define and track [19] and implementation was less universal. Community crisis response to violence and police harassment was established, and access to government entitlements such as identification and ration cards featured in the more comprehensive programmes [10]. Since structural interventions are inherently context dependent and situation specific, local implementers were given the flexibility to adapt programmes to their context, as long as the minimum components were kept universal. Whilst programme activities were first led by ‘non-community members’, the CM process was supposed to lead to the formation of community-based organisations (CBOs), ensuring a transition of programme ownership from the implementing NGO to the benefitting FSWs. The OD activities of leadership training and capacity and network building facilitated this process [10].

Given the large-scale nature of Avahan, the evaluation model portrays a ‘single’ programme irrespective of context, which needs to be tested across settings [10]. While in-depth formative research has systematically documented the implementation of Avahan CM efforts in Andhra Pradesh [6,20,21,22], there is no analysis thus far of the implementation of CM in complex urban environments. The metropolis of Mumbai has been identified as a challenging environment for CM among FSWs [23,24] confirming earlier studies in the 1990s pointing to the significance of a unfavourable political ideological context for collectivisation in Mumbai compared to Kolkata [25,26]. With an estimated FSW population of more than 36,000 [27], Mumbai and neighbouring district Thane are known sex work ‘hubs’, and have been named the ‘epicentre’ of HIV in the Indian context [27]. Here, Avahan complemented efforts already introduced by India’s National Aids Control Organisation (NACO) overseen by Maharashtra State Aids Control Society (MSACS) in Thane and by Mumbai District Aids Control Society (MDACS) in Mumbai. Both Avahan and NACO programmes were implemented through partner (NGOs). When the results of bio-behavioural surveys revealed that HIV prevalence among FSWs in the two metropolitan cities increased from 23.6% to 30.6% between 2006 and 2009 [27,28] understanding how large-scale interventions impact and interact with the sex work industry in a complex urban metropolis became crucial.

This qualitative study explores factors impacting on participation and implementation of community mobilisation interventions (CMIs) among sex workers in the complex socio-economic environment of urban Mumbai and Thane (hereon ‘Mumbai’). Through a thematic analysis of in-depth interviews, group interviews and focus group discussions with programme implementers, we show how CMIs contribute to shaping the social context in which they are implemented. We discuss how the evaluation framework with its requirements to monitor and demonstrate success [10] may adversely affect the risk environment.

## Methods

Qualitative description [29,30] was the methodological approach to this study. Rhodes’ [31] risk environment framework, much applicable to the context of sex work, informed our approach to explore the physical, social, economic, and policy environment of sex work and interventions.

### Data collection

This paper is based on a subset of data from a larger qualitative study that sought to explore and understand the rise in HIV prevalence among FSWs in Mumbai. The study used purposive sampling, starting from an initial list of prospective organisations and individuals representing key constituencies relating to HIV prevention work in Mumbai. Participants were chosen to complement each other in terms of their experience with and connection to Avahan and NACO (MDACS/SACS), geographical location, professional position, and key population which they belonged to or engaged with. Sequential referral sampling was used to allow flexibility and the exploration of emerging themes [32]. The larger study consisted of 36 individual interviews, nine joint interviews, and ten focus group discussions (FGDs) interviewing various stakeholders. Implementation challenges of community mobilisation emerged as an important theme and for this paper we used the 20 interviews where respondents had elaborated on these challenges.

Interviews were audio recorded and subsequently transcribed by members of the research team and external consultants. The interviews conducted in Hindi and/or Marathi were translated into English.

### Study participants

Eleven individual interviews, three joint interviews, and six group interviews formed the data for this analysis (Table 1). In total, we interacted with 63 participants (42 female and 21 male). Apart from one sex worker who was not a peer educator and one previous client (now boyfriend of a bar girl), all participants were involved in programme implementation: 41 at grass-roots level either as peer educators (PEs) or outreach workers (ORWs); 9 program coordinators (PC) at mid-level and 11 senior staff in leadership positions. Implementers’ approaches to HIV prevention differed among study participants. Some worked exclusively with FSWs, others defined their constituents as women, migrants or poor communities, including women who sell sex. Some offered HIV prevention services only, others comprehensive family health services, yet others demonstrated a holistic approach to health and HIV prevention in their work with FSWs, also addressing women’s other pressing needs. Participants’ profiles are presented in Table 1. When using quotes we indicate the participant’s position (e.g. senior staff), affiliation (e.g. CBO), approach (e.g. comprehensive), and interview type and number (e.g. IDI 5), which refers to the information provided in Table 1. All interviews analysed in this report were conducted between March and July 2012.

**Table 1:** Participants profile .

ID number	Type of interview	Participant(s)	Affiliation	Organisation’s target population / representing	Organisation’s remit
**1**	In-depth interview	Male senior staff	Former NACO partner NGO	Migrants	HIV/AIDS [Exclusive]
**2**	In-depth interview (joint 2 pax)	Female senior staff	NACO partner NGO	Clients-migrants	HIV/AIDS [Exclusive]
**3**	In-depth interview	Male senior staff	NACO service provider/health care facility	FSWs: Brothel-based	N/A Service provider
**4**	In-depth interview	Male senior staff	Avahan partner NGO	FSWs: Home-based, bar dancers; Migrants	Health [family health]
**5**	In-depth interview	Female senior staff	Former Avahan partner CBO	FSWs: Brothel-based	FSW [Comprehensive, HIV/AIDS being one component]
**6**	In-depth interview (joint 2 pax)	Male senior staff	Avahan state lead partner	FSWs	HIV/AIDS
**7**	In-depth interview	Male senior staff	MDACS/NACO	All key populations	HIV/AIDS [Exclusive]
**8**	Group discussion 7 pax	Male and female ORWs	Avahan partner NGO	Male migrants; FSWs bar dancers	Health [family health]
**9**	In-depth interview	Female sex worker	N/A: Intervention recipient	FSWs, brothel based	N/A Intervention recipient
**10**	Group discussion 3 pax	Male and female PCs	Avahan partner NGO	Male migrants; FSWs bar dancers	Health [family health]
**11**	Group discussion 4 pax	Female PEs and PC	Avahan partner NGO	FSWs: Brothel-based	Health [family health]
**12**	Focus group discussion 11 pax	Male and female ORWs, PEs and PC	Avahan partner NGO	FSWs: Brothel-based	HIV/AIDS [Exclusive]
**13**	In-depth interview	Male intervention recipient	N/A	Partner of bar girl	N/A Intervention recipient
**14**	In-depth interview	Female PC	Former NACO partner NGO	FSWs: Hidden	Women's issues [Comprehensive, HIV/AIDS being one component]
**15**	In-depth interview	Female PC	Avahan partner CBO	FSWs: Brothel-based, home-based/hidden	HIV/AIDS [Exclusive]
**16**	Group discussion 9 pax	Male and female ORWs and PEs	Avahan partner CBO	FSWs: Brothel-based, home-based/hidden	HIV/AIDS [Exclusive]
**17**	In-depth interview (joint 2 pax)	Female senior staff and male PC	Avahan partner NGO	FSWs: Home-based, bar dancers, floating	FSWs [Comprehensive, HIV/AIDS being a component]
**18**	Focus group discussion 12 pax	Female ORWs and PEs	Avahan partner NGO	Bar girls	FSWs [Comprehensive, HIV/AIDS being a component]
**19**	In-depth interview	Male senior staff	MDACS partner NGO	Injecting drug users	IDUs [Comprehensive, HIV/AIDS being one]
**20**	In-depth interview	Female PE	MDACS partner NGO	Injecting drug users	IDUs [Comprehensive, HIV/AIDS being one]

Abbreviations used: ORW = outreach worker; PC = program coordinator; PE = peer educator; NGO = non-governmental organisation; CBO = community based organization; NACO = National Aids Control Organisation; MDACS = Mumbai District Aids Control Society.

### Data analysis

We conducted a thematic analysis of interview and discussion transcripts.

The dataset was coded using the computer software ATLAS.ti (Version 7.0, Germany). The first stage of the analysis entailed open coding. This method allowed for a systematic but inductive analysis of the data at an early stage in the research. The iterative process of familiarisation, coding, charting and analysis facilitated the constant re-evaluation and interpretation of the emerging data and its meaning. A refined coding scheme based on the open coding, with clear application criteria was further developed in the process. The research team was involved in the analysis of the data, developing the coding schemes and identifying the main themes.

The Institutional Ethics Committee, Tata Institute of Social Sciences (TISS), Mumbai, India and the IRB from London School of Hygiene and Tropical Medicine approved the study. Written informed consent was sought for participation and audio recording of interviews. For group interviews, one person signed the consent form on behalf of all participants in front of the group. All those participating in group interviews and discussions were requested not to mention names of individuals to protect identities. All consent procedures were evaluated and approved by the IRB and all data have been password protected with access restricted to research team members and associates only. In this manuscript, we made every attempt to protect research participants’ identities when using quotes.

## Results

We identified 6 interlinking contextual themes that impact community mobilisation efforts among women who sell sex in Mumbai. These were: diversification of buyers and sellers of sex; stigma and identity; management and control of sex work (includes efforts to control and police sex work from both madams/pimps/brokers, interventions, police/society); sex workers’ mobility; a heterogeneous sex worker population; and the programme model and – ownership. Themes are presented in Table 2 alongside representation of interviews in which they featured.

**Table 2:** Factors identified to impact on participation and implementation of community mobilisation interventions (CMIs) and interviews in which they featured.

Factors impacting participation and implementation of CMIs	Theme discussed in interview:	Illustrative quote
**Diversification of buyers and sellers of sex (hidden sex work)**	1,2,3,4,5,6,7,10,12,14,15,16,17,18	“Where they do sex work will change. We have seen that steadily the number of sex workers in brothels has declined remarkably. And bar based, home based and floating sex work has increased exponentially. So this is going to take place. Our outreach will have to change accordingly as the changes happen”(7)
**Stigma and identity**	2,4,5,6,7,8,10,12,14,15,16,17,18,19	“However we try to convince them they do not trust us easily. Even in our repeated visits they continue to deny that they are sex workers.” (18)
**Management and control of sex work**	1,3,4,5,6,7,8,9,10,11,12,15,16,	“They do not allow the new girls who are under their debt to come out.” (16)
**Sex worker mobility**	1,2,3,4,5,6,7,9,10,11, 12,13,15,16,17,18	R: Some go on contract basis. I: What is that? R: They have contract of 15 days but will work for 17 days for which they get 25–30000 rupees. (…) They are taken to Mumbai, Pune, Nasik, Bangalore, Hyderabad, Delhi, Goa and many such big places. I: How many times does a girl make a contract? R: It depends upon her financial need. Some go regularly, some 2 or 3 times in a year. (13)
**Heterogeneous FSW population**	1,2,3,4,6,9,10,11,12,15,16,17	Competition is also increased. Like other labour charges have gone down because of migrants from other states same way because of Bangladesh’s girls' sex workers rates are also gone down. They will do anything in cheaper rate. So the number of customers of local sex worker’s is also reduced. (12)
**Programme model and ownership**	1,2,3,4,5,10,11,12, 14,15,17,19	Sometimes targets to be met are so many because of which quality gets compromised. (10)

The findings demonstrate how the identified factors influence both participation and implementation of the CM interventions and reveals close interactions between the interventions and the sex industry.

### Hide and seek: Diversification of buyers and sellers of sex

The participants expressed that the organisation of sex work was changing, and that sex work was becoming more “hidden”. “Earlier sex work was concentrated in brothels,” said one senior staff (7), who further explained “there is definitely a shift in typology from brothel-based to street-based, phone-based and even home-based”. The more affordable brothel-based FSWs were becoming less in demand, whilst home-based, bar girls and floating FSWs experience more popularity. Other participants confirmed: “Brothels are routine. Customers want something different” (former client (13)) and explained that relaxed sexual norms and attitudes contributed to a change in clients’ preferences for providers and the emergence of a diverse population of women selling sex.

*“So spending power of people has increased and need of the sex & sexuality is also changed. They want to go to the cleaner place (…) people did not want to go to the red light area where the rates were cheaper and the place was not clean.”*

*PC, NGO (comprehensive) (IDI 17)*

The ‘conventional’ brothel and street based FSWs are the easiest to locate for interventions: highly visible, with high transaction frequencies and rates affordable to labourers and migrants, they are less desirable for clients who can afford to pay more to avoid the associated stigma and/or HIV risk. The bar girls cater to a different segment of relatively wealthier clients. While it is commonly known that some ‘bar girls’ offer sexual services, they will often deny being in sex work (PC (10)). After the ‘bar dance ban’(dance bars were banned in the state of Maharashtra in August 2005, with the passing of an amendment to the Bombay Police Act 1951; the ban was revoked in 2013), they lost their livelihoods as dancers, and working as waitresses they are more likely in need of supplementing their income by selling sex. While interventions can still reach them at bars, other women selling sex — either on regular or sporadic basis — are more challenging to identify: including secretive housewives, poor construction workers, and local flower or vegetable vendors.

*“Now a day many residential women are getting involved in the sex work. There are women who work in handlooms under Mehta [supervisor]. There are rag pickers. Residential women involved in sex work are in office, in chawl [cramped small one/two room residences]. They are available at every place in the area.”*

*ORW, NGO (Health) (GD 8)*

Continuous targeting of FSWs was said to create an awareness of the link between conventional sex work and HIV in the general population, and moulded clients’ perceptions about who is at risk, who is safe or ‘clean’ and thus preferable for sexual relationships. The intervention has thus – unintentionally – contributed to creating demand for a whole new hidden market of sex work.

*“If you see the trend which is changing when it comes to sex work, again credit goes to HIV intervention, the term “Targeted” itself is such negative term. The entire messages about HIV were based on fear from the beginning. They were threatening messages about fear to contract HIV and so the entire focus was: the reason why men are getting HIV was because they go to sex workers.”*

*Senior staff, CBO (Comprehensive) (IDI 5)*

The ORWs (16) confirmed this perception and explained “known brothels are stigmatized because of HIV. Customers consider home-based women as 'good women' and go with her in a lodge, because she is not labelled as a sex worker. Therefore the number of customers coming to brothel has reduced”. This change in clients’ preferences may reinforce FSWs reluctance to participate in or be associated with the interventions. ORWs were prevented from visiting women’s houses or brothels, as “they [FSWs] will lose their customers” (ORWs (16)).

With new forms of solicitation, women who sell sex can avoid traditional ‘hotspots’. The availability and affordability of mobile phones reduce the need for personal and visible solicitation, further supporting the trend of ‘hidden sex work’. One programme coordinator expressed: “Nothing is happening without a mobile (phone), some women have expensive mobiles gifted by customers, which ensures “smooth” communication between client and provider.” (PC (10)). Using mobile phones for solicitation and client contact allows women to build their own networks.

The on-going diversification of sex work beyond the brothels and streets towards less visible, less networked and seemingly more autonomous forms of sex work creates an increasingly difficult environment for HIV prevention programmes to reach out to women selling sex, let alone mobilise them.

*“All these hidden populations e.g., home-based sex workers can easily get influenced by money and provide sex without condom. You cannot reach out to this hidden population to teach them negotiation skills.”*

*Senior staff, Health care facility (IDI 3)*

### Label me not: Identity and stigma

Categorizing FSWs by place of solicitation seems to have lead to the false assumption that a woman’s identity is closely linked to her occupation as a sex worker. It seemed women who sell sex will not necessarily adopt ‘FSW’ as their (main) identity, and most will indeed resist a label experienced as stigmatising.

*“However we try to convince them they do not trust us easily. Even in our repeated visits they continue to deny that they are sex workers.”*

*ORW, NGO (comprehensive) (FGD 18)*

Brothel-based FSW could not avoid being publicly identified as sex workers and as a consequence “they have lost their respect in the community …” (ORWs (16)). However, other beneficiaries’ unwillingness to accept the limited identity of ‘sex worker’ was well understood by programme managers, who recounted the challenges of sensitising field staff

*“[W]e said you cannot call everybody a sex worker. There are housewives and construction workers only doing sex work part time. At our level we are very clear. But then we are training implementers on the ground, and 2nd and 3rd level [who might not internalise such clarity]. … the message that comes through is that ‘all are sex workers’. You have labelled them.*

*Senior staff, NGO (HIV-only) (IDI 2)*

There were reports on clashes between the interventions’ monitoring needs and the needs of the beneficiaries to remain anonymous. The same senior staff (2) explained how evaluators representing the funding agencies verify programme monitoring and visit to check that they are “really working with sex workers”. Often, it was reported, recent graduates in junior bureaucratic positions lacked the necessary contextual experience to understand ground realities.

*“Evaluators are trained to say “Are you a *dhandewali* [reference to sex worker with utmost derogation]?” Women get offended. ‘Why should you call me a ‘*dhandewali*’? I am a woman first’. And so recently one evaluator at one site, in the evaluation recorded zero marks because women had said no, I am not a sex worker.”*

*Senior staff, NGO (HIV-only) (IDI 2)*

In Avahan’s Astha Gats [groups] programme service provision and referrals were delivered upon ‘risk profiling’ of FSWs and ‘micro planning’ of activities.

*“We have a protocol for all the peer educators saying that within 7 days of their [new FSWs] registration, they have to be getting one SBC [Strategic Behaviour Change] Communication session, they have to be brought to the clinic, they have to get condoms according to the frequency they have [sex]. And recently we have introduced HIV testing also. So this is the protocol which has been followed (…) our data shows 98 per cent of our population who have entered our area, new, are covered within the span of 7 days.”*

*Senior staff, SLP (IDI 6)*

While the programme implementer attributed success in coverage to this micro-planning and risk profiling strategy, participants with more ground-level experience emphasised the challenge of attempting to discuss highly intimate issues with women before trust had been established, which may question the validity of self-reported risk behaviour. Additionally, and although unintended, the intervention may be stigmatising women further by classifying them according to ‘risk behaviour’ and number of clients.

*“We first ask [women] about sex [sexual activities] and do the registration. Like that our intervention starts. Who would like to talk about their sexual activity in the first interaction?”*

*PC, NGO (Health) (GD 10)*

Women who are newly enrolled in programmes, who thus acknowledge selling sex may still deny long association with sex work. The ORWs (16) explain how the stigma affects what women tell them: “They [FSWs] tell us ‘I am new; I started sex work one month back, two months back.’ They lie to us. When they test [HIV] positive we realise that they were already positive before coming here.”

The persistent labelling seemed to have amplified the pre-existing stigma and made some women shun the programmes. Certain organisations seemed to show more flexibility – and sensitivity – when reaching out to ‘hidden’ or undisclosed sex workers, highlighting the need for building rapport with the women. In particular this sensitivity seemed to apply to NGOs/CBOs who were running comprehensive projects in an area prior to the roll out of Avahan and NACO-funded targeted interventions. Their established presence and contextual understanding enabled holistic approaches, where HIV prevention services could be integrated into the organisation’s general community health outreach. Hence they could deliver non-discriminatory and non-stigmatising services *also* to women who sell sex. A programme coordinator (14) explained how they integrated FSWs in their general work: “women did not know that other women coming for meeting are in sex work or not, as they were home based sex workers; so we did talk about condom use etc.—but in generalized way. None of the interventions started directly on issues of HIV/AIDS”. Outreach workers (16) confirm that “We take at least 4 months to interact with home based KP [member of the key population]”. Some organisations demonstrated a wider interpretation of whom the ‘community’ consisted of, especially among those who were working with home-based or ‘hidden’ FSWs.

*“We were not working with this group [FSWs] as a separate group. It was an integrated approach. Meetings used to be together and not in isolation. Some peers were from sex workers and some from other community. Our staff knew internally that the focus is on sex workers but there was no separation. In our health camps under Avahan project, women from general population were also receiving services with sex workers so there was no taboo.”*

*PC, NGO (Comprehensive) (IDI 14)*

This community approach opposes maximum coverage – one of Avahan’s main objectives. The explicit strategy by the NGO to avoid programme-induced stigma by not openly/outwardly expressing that FSWs were a target population is far removed from the requirement of public disclosure of a sex worker identity—an integral step on the empowerment pathway in the community mobilisation framework [19]. Nevertheless it seemed vital to facilitate outreach.

### Power play: Management and control

Various stakeholders continue to control FSWs workload, exposure to interventions and location. The majority of FSWs whom the interventions were targeting seemed to be under the control and management of a ‘pimp’ or ‘madam’ (for simplicity the term ‘pimp’ is used when referring to the concept of a person being in control of, or ‘owning’, the sex worker including: gharwalis/brothel keepers/brothel owners (‘madams’), pimps, agents, and boyfriends and husbands acting as agents.). These were reported to constitute a major barrier for ORWs and PEs to access FSWs, as they want to keep certain girls hidden from the interventions. The level of ‘management’ depends on the woman’s age, debt, and perceived profitability. For instance, to avoid new girls running away and the ‘rescue and rehabilitation’ of minor girls, they are kept locked up in a brothel or at other places in the suburbs. Either way, it seemed to make outreach and CM among this group inconceivable.

*“They keep the new girls, whether minor or adult, at the outskirts of city where they have their own houses for 3 to 6 months. When these girls get used to sex work, they are brought to the red light area.”*

*Senior staff, CBO (Comprehensive) (IDI 5)*

Similarly ORWs (16) explained that “new girls who are under their [madams/pimps] debt” are not allowed to “come out” from their rooms. In an effort to overcome this barrier, PEs and ORWs attempted to build rapport with pimps. Some strategies involve merely persistence and persuasion, the message being that healthy sex workers attract more clients and would thus benefit the pimp. A senior staff (7) revealed that they “have to work through them [pimps]. Because ultimately if we are able to convince them that it is for their benefit, it is always good”. A PE (12) explains the difficulty of gaining trust: “we tell them we are also sex workers like one of you, running this CBO. But still thing does not enter in their mind. They hide the girls.”

The fragile relationship between the intervention and the pimps is further compromised by another powerful stakeholder: the police. FSWs are subject to police harassment and persecution, and the imminent threat of police raids keeps both FSWs and brothel owners wary of risking exposure. The ORWs expressed frustration over their limited power and influence when faced with the shutting down of brothels after raids, which subsequently put the FSWs at increased risk.

*“That time their priority is also to earn money and not to get condoms. As an NGO they demand from us, to intervene and release them from police, but we are not getting money to do such work. If we do not help them in such problems then even they do not entertain us [give access]. When they restart their business and we go to them, they say we do not need your condom and doctor services. We can get it from private doctors. You did not help us when we were in problem. We do not need you now.”*

*ORWs,NGO (HIV-only) (FGD 12)*

Lack of trust in the ORWs and PEs in some cases led pimps and FSWs to believe that the intervention was cooperating with and informing the police, bringing on the raids as demonstrated by a PE (12): “Some time it has happened that we took guest for round in the area and after half an hour raid took place. So immediately they link it with our visit.” Hence access to interventions was perceived to jeopardize the incomes of both pimps and FSWs.

Intervention strategies included police sensitisation and relationship building between the programme and local police officers as noted in FGD with ORWs and PEs (18): “We have good relations with police, we tie *rakhis* [silk threads, symbolic and significant gesture to douse hatred across communities] to at least 50 police-men on *rakshabandhan* [a Hindu festival]. Whole staff of [the organisation] go to the police station and some women from our community also accompany if they want to.” Another structural component in the interventions was the establishment of community crisis response groups that offer support to FSWs when faced with prosecution or police violence. While these are important steps, programmes were unable to prevent raids enforced under the India’s Prevention of Immoral Trafficking Act (PITA), and ORWs (12) complained that pimps “cannot even understand the difference in the organisations which are working for police and organisation which are working for health, nutrition etc.” Building adequate alliances with pimps and police was not achievable in a context where multiple organisations and agencies—often representing competing interests – intervened simultaneously in one particular area.

### On the move: Sex workers' mobility

FSWs mobility was linked to aspirations to earning more and avoiding police raids. Some FSWs are shifted by the pimp or brothel keeper to avoid raids or shut downs, others take on relatively well-paid contracts for weeks at a time in other sites.

*“(…) they go on contract to Poona, Bangalore for 17 days, 20 days or one month. They get 30,000 rupees. The rate is fixed based on the girl’s looks, how many clients she can take and what is her age. Contractor should also be able to make the profit. If girl takes four clients in daytime and one in the night then she gets 30,000 rupees. Half of the money she gets before going on contract and half after she completes the tenure.”*

*PE, NGO (Comprehensive) (FGD 18)*

Some FSWs also travel independently to community festival sites in search of clientele. Continuous mobility clearly challenges service provision and follow-up leaving little time to achieve behaviour change within a community (senior staff (6)).

*“[T]he mobility is very high. They come on contract of one to 3 months. By the time we get their HIV-test result they are gone from the area without collecting report. Sometimes we are able to give their report and when we go for the next visit for follow up we come to know that they left the area.”*

*PC, NGO (HIV-only) (FGD 12)*

Participants saw mobility as an insurmountable barrier beyond their control. Indeed it seems to confirm the incompatibility of the context with community mobilisation efforts.

### Come together: Mobilising a fragmented community

The FSW-population in Mumbai is highly heterogeneous. Women migrate to Mumbai for work from across state, nation and countries, bringing with them a mix of cultures, languages, notions of sex and sensuality, and appearances. As such the FSW-population reflects Mumbai’s multicultural population, which, in the Indian context presents a unique intervention setting. A senior staff (6) described it as an uncompetitive environment where “Sex workers [are] catering to different segments of the population in terms of their income … culture, language and background”. All other respondents provided perspectives that contradicted this view, with anecdotes and opinions that represented competition and conflicting interests between FSWs. For example, another senior staff (2) explained that in her area of work, Bengali FSWs are competing for the same clients as the local FSWs, but are considered prettier and charge lower rates, which make them fetch more clients. Local brothel-based FSWs confirmed the beauty and attraction of Bengali and Nepalese girls, but contradicted the question of their cheap rates. Yet the rivalry creating mistrust and animosity between the FSWs is evident:

*Respondent 2: “Bengali & Nepali has got more rates because they are fair in colour, uses short pants, will put goggles. We wear sari & blouse. … For money we do not want to sell our honour.”*

*Respondent 2&4: “Nepali also has more [HIV] positivity. Positivity is more among the good-looking girls. It is less among those who look ugly”*

*PEs, NGO (Health) (GD 11)*

FSWs seem to stigmatise their peers by assuming they are HIV positive, and by ascribing ‘inappropriate behaviours’ like non-conventional (i.e. non-vaginal) sexual services, revealing outfits, and compromised condom use to other categories of FSWs. Badmouthing their competition especially ‘lower’ forms of sex work than their own, women were said to attempt upholding their own status and popularity.

*“Bar girls do not go on street or in brothel. They consider it inferior. They say that ‘we do not do that dirty work’.”*

*PC, NGO (Health) (GD 10)*

This apparent hostility emerging from within the ‘community’ of women who sell sex may represent another challenge to CM. Most programmes worked with only one segment of the FSW-typology, attempting to create community based groups (CBGs) among women who worked on the same street or in the same brothel or bar. A ‘natural coherence’ sometimes stemmed from a brothel owner’s inclination to recruit/employ women from their cultural background, facilitating collectivisation at community group-level.

*“[L]argely the process was that within a small, concise geographical location which captures one type of sex work; you will have less heterogeneity or less diversity. (…) So we came up with this concept of having centred groups, ranging from 8 to 12, 15 or even 20 sex workers. Depending on what specific type of sex work or the specific location, within this group you will have much more homogeneity. And the homogeneity may not be in the type of perhaps the cultural background, but at least in terms of the type of sex work (…) The next step then is to bring some of these groups together, (…) and then they form a CBO (…) and then all these CBOs come together to form a federation or an umbrella organisation. So because of this process, it gives us this confidence that we have a bottom-up kind of approach. Now there is a lot of cohesion between each of these groups.”*

*Senior staff, SLP (IDI 6)*

The respondent’s formulaic presentation of CBO formation to some extent disregarded the significance of FSWs’ different cultural or ethnic backgrounds in the initial group formation, focusing solely on FSW-typology. Additionally, it remained unclear what factors exactly led the FSWs within a collective to overcome the mistrust and rivalry caused by their ‘heterogeneity’, or how aggregating smaller groups would overcome a lack of cohesion. The lack of unity and support among diverse FSWs in Mumbai made mobilisation unrealistic to many other respondents, drawing comparisons with the success of Sonagachi:

*“If you take Calcutta as a model in to consideration… same thing we are doing everywhere, but the results people are getting in Calcutta are different than what we are getting here. Because if you go to Calcutta’s red light area, at Sonagachi, you will find very good cohesive group, most women are from the local areas, they speak a common language.”*

*Senior staff, CBO (Comprehensive) (IDI 5)*

The short timespan in which the interventions attempted CBO formation added to the constraints.

*“This thing will not happen within a year, two years of intervention. Getting all thousand women tested in a year two times is possible, but getting these thousand women to sit together and discussing the issue is a process. And that process is going to take time. And I don’t think that anybody have that patience, for that process is really long drawn.”*

*Senior staff, NGO (HIV-only) (IDI 2)*

### Take lead: the programme model and ownership

Performance targets and monitoring of programme indicators are central to the business approach adopted by Avahan, and implicit in the national guidelines (NACP-III). Implementers reflected on difficulties reaching targets in a changing context.

*“When we were working with home based sex workers, the indicators which are used with other group, of target, condom use, ICTC testing, were very difficult to reach. Here you have to work per case, you don’t find a group of women as you will find with brothel based or bar girls. We could not do direct intervention like other people [who are working with conventional typologies] and so our figures used to be low.”*

*PC, NGO (comprehensive) (IDI 14)*

Despite their acute awareness of a shifting trend in sex work, the inherent pressure to demonstrate performance was reported to skew the programme towards the ‘easiest to reach’ and ‘most visible’ population of sex workers commonly found in brothels, bars, and on the streets. With the need for measurable outputs and outcomes, indicators begin to determine programme content and shape service provision. Condom use as proximate determinant of HIV transmission has been at the centre of both programme outputs (condom messaging and distribution) and impact evaluations (self-reported condom use). Condom use was thus not surprisingly a recurring theme in all interviews, and the narratives followed a similar pattern. First were claims of the success of the intervention, expressed as “Now-a-days, sex workers are not doing sex without condom. They refuse to do it without condom even if more money is offered to them. (…) Condom is available everywhere” (ORW (8)).

However, the inability to respond to either the beneficiaries perceived needs or the realities on the ground was also a common theme. Frustration was expressed about the entire focus being on HIV only:

*“it is one of the 100 issues” [that FSW face in this dynamic context] (…). We are so focused on HIV because of which quality gets suffered. We should think about other issues too but we are only about completing targets and not really having comprehensive approach.”*

*PC, NGO (Health) (GD 10)*

Lack of funding and mandate for the follow-up of HIV positive women proved a particular barrier to attracting and keeping beneficiaries. In contrast, implementers who offered comprehensive and holistic services not solely targeting HIV-prevention or FSWs reported more positive experiences with CM. These services were typically delivered by NGOs/CBOs with long-standing presence in the community, who used Avahan funding to supplement existing services.

*In our experience it [collectivisation] is not impossible. But it is required that core group has to be sustained, paid and supported. We have taken these women in groups of 30–35 to police station, to social service department, to bank etc. (….) Our women go to police station and they are treated very well, this kind of network, atmosphere, linkages work well. 28 women from this project were selected for committees and they are performing very well.”*

*Senior staff, NGO (Comprehensive) (IDI 17)*

According to one senior staff (5), this support was lacking for newly created CBOs, to which implementing NGOs were transferring ownership under Avahan’s CM programme model: “Most of the CBO formation, this trend that started is very superficial, they will not get rooted here. Most of the CBOs have remained on paper because formation of CBO itself is a huge process, you can’t form a CBO just like that”.

While performance indicators seem to indicate that elements of this transfer process are underway, ground realities seemed quite different. Our participants were sceptical about the sustainability of these community organisations post-Avahan, and were convinced that “their real face” (senior staff (5)) will be shown during the transition of the Avahan initiative to the Government and the CBOs. Another senior staff (2) doubted the government had the capacity (or inclination) to take on this component of the model and feared that “Now is the time to engage more of the sex workers, and that is when most of the programmes are coming to an end”.

## Discussion

Our data demonstrate that large-scale HIV prevention programmes, which included community mobilisation efforts, have been affected by and interacted with a rapidly changing context in Mumbai and Thane. The narratives of some senior managers detailed the implementation of programme goals underlying the CM evaluation framework, framed in the language of Avahan’s theory of change. However, this simplicity was contested by the ground-level staff revealing the reality of very complex social dynamics that did not easily slot into the programme framework logic. We review our findings according to Avahan’s three community mobilisation processes (identification with other sex workers, collectivisation and taking ownership of the intervention) [10], showing how targeting and monitoring performance may have intensified contextual challenges.

The programme theory for CMI hypothesises that participation in intervention activities leads to identification with others: the first step of the CM process. A crosscutting finding in our data-and a major challenge for the programme- is the strong motivation of many women *not* to identify as sex workers. Clients’ preferences effectively changed toward women not openly disclosing as FSWs. In competition for clients, remaining ‘incognito’ thus became a rational, financial decision for FSWs. Targeted interventions draw attention to the link between FSWs and HIV risk. It is difficult to prevent safer sex messages being reinterpreted as a way of communicating blame and risk of HIV to sex with FSWs. Scale-up of targeted interventions thus unintentionally reinforces sex worker stigma [33]. The programme practices of registration and monitoring-all imperative to Avahan’s strong information systems- were experienced as stigmatising too, further feeding women’s persistent denial of being ‘a sex worker’, and indeed increasing challenges for outreach (let alone mobilisation). In contrast to Sonagachi [12], it was reported women did not find the ‘sex worker identity’ empowering. Rather, they took offense and were trying to avoid it. Research in Andhra Pradesh also showed that the public ‘label’ of being a sex worker CBO effectively made some women reject the clinical services to shun the association to sex work [9].

We demonstrated that the highly diverse population of FSWs in Mumbai and Thane is not a ready-made ‘community’ to mobilise. Many FSWs are migrants from other states and countries, speaking different languages and competing for the best paying clients. The rivalry and lack of solidarity is not uncommon and a known obstacle for community mobilisation [34, 35]. The already heterogeneous ‘community of FSWs’ in Mumbai has become increasingly dispersed and hidden. Selling sex outside the known hot spots forced, and enabled, more ‘hidden’ ways of solicitation as well as the rejection of the label ‘sex worker’.

The dispersal and diversification of sex work not only impedes the process of ‘identification with others’ but also obstructs the second step in the CM process: collectivisation. Through increased social cohesion and a resulting sense of collective identity, the programme theory expects FSWs to ‘invest emotionally in the large entity’, tackle their problems and advocate for themselves [10]. The programme rhetoric seems clearly at odds with women’s reality in Mumbai, implying a divide of the target population: those who identify with, and are willing to participate in the interventions, and those who will not. A 2010 survey, restricted to sex workers currently registered with the Avahan project in Mumbai and Thane, showed that 71% reported medium or high collective efficacy defined as ‘the belief in the power to achieve goals and address problems together’ [34]. In the 2009 IBBA, collective agency (having attended a public event where one could be identified as a sex worker) was indeed significantly associated with membership of a community group, yet only 5% of sex workers reported being members among this representative sample of brothel and street-based FSW in Mumbai and Thane [36]. Other studies in 2007 and 2008 also observed the lack of community cohesion among FSW in Mumbai [24,37] confirming women’s reluctance and/or inability to participate in CMIs. In Andhra Pradesh too, participation in CMIs was strongly associated with a willingness to be identified in public [20] and diversity among sex workers made collectivisation more challenging [38]. The inherent assumption of pre-existing cohesion among a ‘community’ of marginalised women, which simply needs stimuli to be ‘mobilised’, is clearly false.

Structural activities initiated to support the collectivisation process depended on the communities’ interactions and relationships with other stakeholders. While respondents did claim some success in sensitisation and building symbolic relationships with the local police (like tying ‘rakhis’), the intervention had little control over police raids instigated to either strengthen gentrification or abolition efforts. The mistrust of FSWs, brothel owners and pimps, fostered by the frequent police raids extended to peer educators and outreach workers, suspected of cooperating with the police. Hence these gatekeepers did not allow access to “their sex workers”, controlling FSWs’ exposure to interventions, and acting as a significant barrier for mobilisation of FSWs in this and other contexts [8,34,35]. Stakeholder engagement was poor and Avahan’s programme theory and monitoring framework [10] does not feature a strong component of building community support within FSWs immediate environment. The intention of addressing structural barriers seemed also in conflict with the demands of strong information systems for monitoring the implementation of a standardized Avahan package. Avahan’s success has been primarily measured by demonstrating intervention coverage to saturation level [39]. Based on the implementers’ ability to report on services, structural components got de-prioritised over those focused on individual behaviour change which are easily monitored and measured.

Social dynamics played out at higher structural levels in a metropolis, where a changing political economy has resulted in the gentrification of the traditional brothel areas, exacerbate the already difficult task of transforming power relations within the immediate environment of sex workers. Detailed ethnographies of Sonagachi [8, 40] illustrated the complexities of altering multiple power-relations between the stigmatised FSWs and dominant groups in the community. This process required substantial direct input from non-sex worker public health and development professionals intervening on sex workers’ behalf with gradual and incremental progress to community leadership [8]. These experiences from Sonagachi lead us to question how realistic the ambition of scaling-up community ownership as final stage in the Avahan CM process is. The success of ‘ownership’ is signified by the formation of a CBO with the power and responsibility of HIV prevention initiatives transferred to the FSWs. The MIS measures the ‘dose’ or strength of CM and community ownership by the level of ‘active representation’ by FSWs in programme committees and leadership teams [10]. When ownership is mandated by the requirements of monitoring performance, CBOs become service providers with quotas to fill [41], far removed from bottom-up community initiatives.

A fundamental assumption underlying FSWs taking ownership of the intervention is that sex workers in Mumbai are interested in coming together to serve ‘the public health imperative’. The relentless emphasis on condom use seemed to preclude the programme from taking the perspectives of its beneficiaries into account, symptomatic of a prescriptive solution to a pre-defined ‘community’ need. In Mumbai, but also elsewhere [42,43,44,45], women who sell sex are usually less concerned with the distant threat of HIV than their day-to-day challenges such as poverty, extortion, stigma, social alienation and harassment. Mobilising around HIV has become the aim of CM under Avahan, different from bottom-up CM initiatives of a self-directed nature [42]. In this conceptualisation of CM too much responsibility is put on sex workers themselves to prevent HIV. Relying on members of marginalised groups to lead HIV prevention too early on is not realistic [8]. Funding agencies have high and contradictory expectations from CBOs demanding them to be both community-led and bureaucratically and managerially savvy [41]. Nambiar’s [46] documentation of the engrained and institutionalised stigma towards FSWs and people living with HIV among health care providers, NGO workers and ‘peer’ educators alike, remind us of a historical context in which the discriminated FSWs have little reason to trust NGOs, and PEs, despite claims of egalitarianism.

Whether full ownership of HIV prevention by a community of sex workers is ever feasible and compatible with maximum reach is questionable. In Mumbai, strong community groups creating enabling environments that reduce vulnerability and risk and increase service coverage and quality, as hypothesised in Avahan framework [10] seem unlikely. A more pertinent question may now be how to reach women in sex work with HIV prevention. It behoves us to discuss the successes in reaching hidden groups of women selling sex in Mumbai. Some established NGOs/CBOs avoided intervention-induced stigma by foregoing the registration of home-based FSWs as ‘sex workers’. Within Avahan, local implementers were granted this ‘flexibility’ to adapt programmes to their context [19]. These explicit strategies to avoid stigma were not rapid and they certainly did not lead to community mobilisation. However, they did facilitate a comprehensive and patient approach to community health, protecting some of the most vulnerable women. This flexibility granted by Avahan could only be translated into sensitive programming by NGOs/CBOs with a more comprehensive health and development remit. In contrast, newer organisations whose funding was exclusively for STI/HIV prevention lacked the know-how and resources to divert from the prescriptive design of the intervention. The gap between the ground realities in Mumbai and Avahan's theory of change reveal that CM cannot be achieved short-term. Both continuous context-responsive adaptation responding to the priorities of the community [47] and building ‘receptive social environments’ [48] seem essential for CM to be health-promoting. Without attention to these, we may well be at the risk of producing harm.

We acknowledge some important weaknesses to this study. First, the research was designed to examine the context of sex work in Mumbai and not specifically to evaluate the scope for CM. The need to explore the contextual constraints for CM emerged during data collection and was warranted by the evidence gap in this area. Second, participants were interviewed about their experiences and observations of the sex industry, including changes in context over time. Since several had been involved in HIV prevention for many years, working for various organisations and/or funders, our ability to differentiate between specific programmes or organisations was at times limited. Every attempt to verify information and anecdotes were made with reference to the data and available literature. Finally, a relatively small sample size may mean that we have not captured all available knowledge and views among the plethora of stakeholders in Mumbai’s HIV prevention and sex work industry, despite relative consensus among our participants.

## Conclusion

Women who sell sex in Mumbai continue to have increased vulnerability and risk to HIV despite intense prevention programming. The changing context is challenging the reach, collectivisation efforts and sustainability of interventions. There is an urgent need for sensitivity in both programming and monitoring, and a renewed understanding that not all women who sell sex are – or want to be – ‘sex workers’. CMIs are not able to reach women who do not want to be identified as FSWs, which in Mumbai, appears to be a growing population. Consequently, the empowerment approach of identifying women as FSWs appears to contradict maximum reach principles. The respondents’ experiences paint a picture of a context in which funders must be willing to provide services based on FSWs actual needs (which far exceed STI/HIV screening and treatment), forego some of the demands of monitoring and reporting, and be willing to continue supporting both NGOs and CBOs.
